# Dendrobine’s mechanism: alleviating cholesterol metabolism disorders via FXR/LXR modulation in NAFLD mouse livers

**DOI:** 10.3389/fphar.2026.1875005

**Published:** 2026-10-02

**Authors:** Zhi Chen, Yi Luo, Yanzhe Xu, Jiaying Li, Zaishuang He, Yichen Gao, Jianyuan Liu, Di Wu, Lin Qin, Daopeng Tan, Yuqi He, Yanliu Lu

**Affiliations:** 1 Key Lab of the Basic Pharmacology of the Ministry of Education, Zunyi Medical University, Guizhou Engineering Research Center of Industrial Key-technology for Dendrobium Nobile, Zunyi Medical University, Zunyi, Guizhou, China; 2 Guizhou Engineering Research Center of Industrial Key-technology for Dendrobium Nobile, Guizhou Engineering Research Center for Orchid Medicinal Plant Breeding and Efficient Application, Zunyi Medical University, Zunyi, Guizhou, China

**Keywords:** bile acid, dendrobine, Dendrobium nobile Lindl, farnesoid X receptor, liver X receptor (LXR), non-alcoholic fatty liver disease

## Abstract

**Background:**

Cholesterol homeostasis is tightly regulated through hepatic synthesis, intestinal absorption, and biliary excretion, with bile acids serving as critical regulators of cholesterol catabolism. Dysregulated cholesterol metabolism and bile acid imbalance are hallmarks of non-alcoholic fatty liver disease (NAFLD), contributing to hepatic lipid accumulation and disease progression. Dendrobine (DNE), a bioactive alkaloid from *Dendrobium nobile*, has emerged as a potential therapeutic candidate for lipid metabolic disorders.

**Methods:**

C57BL/6J mice were fed a high-fat diet to establish an NAFLD model, followed by DNE administration. Hepatic steatosis and cholesterol deposition were evaluated *via* histology and biochemical assays; bile acid profiles were quantified by LC-MS/MS. Gene and protein expression were analyzed using RT-qPCR and Western blotting. *In vitro* studies using cholesterol-laden HepG2 cells further explored DNE’s mechanisms, validated by specific pharmacological agonists and inhibitors of the Farnesoid X receptor (FXR) and liver X receptor (LXR) pathways.

**Results:**

DNE significantly ameliorated hepatic steatosis and reduced cholesterol accumulation in HFD-induced NAFLD mice. LC-MS/MS analysis revealed that DNE substantially expanded the bile acid (BA) pool in the liver, bile, and ileum. Mechanistically, DNE comprehensively modulated cholesterol absorption, biotransformation, and transport by modulating the FXR and LXR signaling pathways. It robustly upregulated key genes involved in BA synthesis (e.g., *Cyp7a1*) and efflux, while downregulating transporters responsible for BA and cholesterol reuptake (e.g., *Ntcp*, *Npc1l1*). *In vitro* validation using specific pharmacological agonists and antagonists further confirmed that DNE upregulates *CYP7A1* and promotes BA synthesis *via* a dual FXR/LXRα-dependent regulatory network.

**Conclusion:**

Our findings suggest that DNE modulates cholesterol homeostasis and alleviates NAFLD in conjunction with BA synthesis, transport, and absorption, in a manner associated with the FXR/LXRα regulatory network, presenting a promising therapeutic strategy.

## Graphical Abstract

**Figure:**
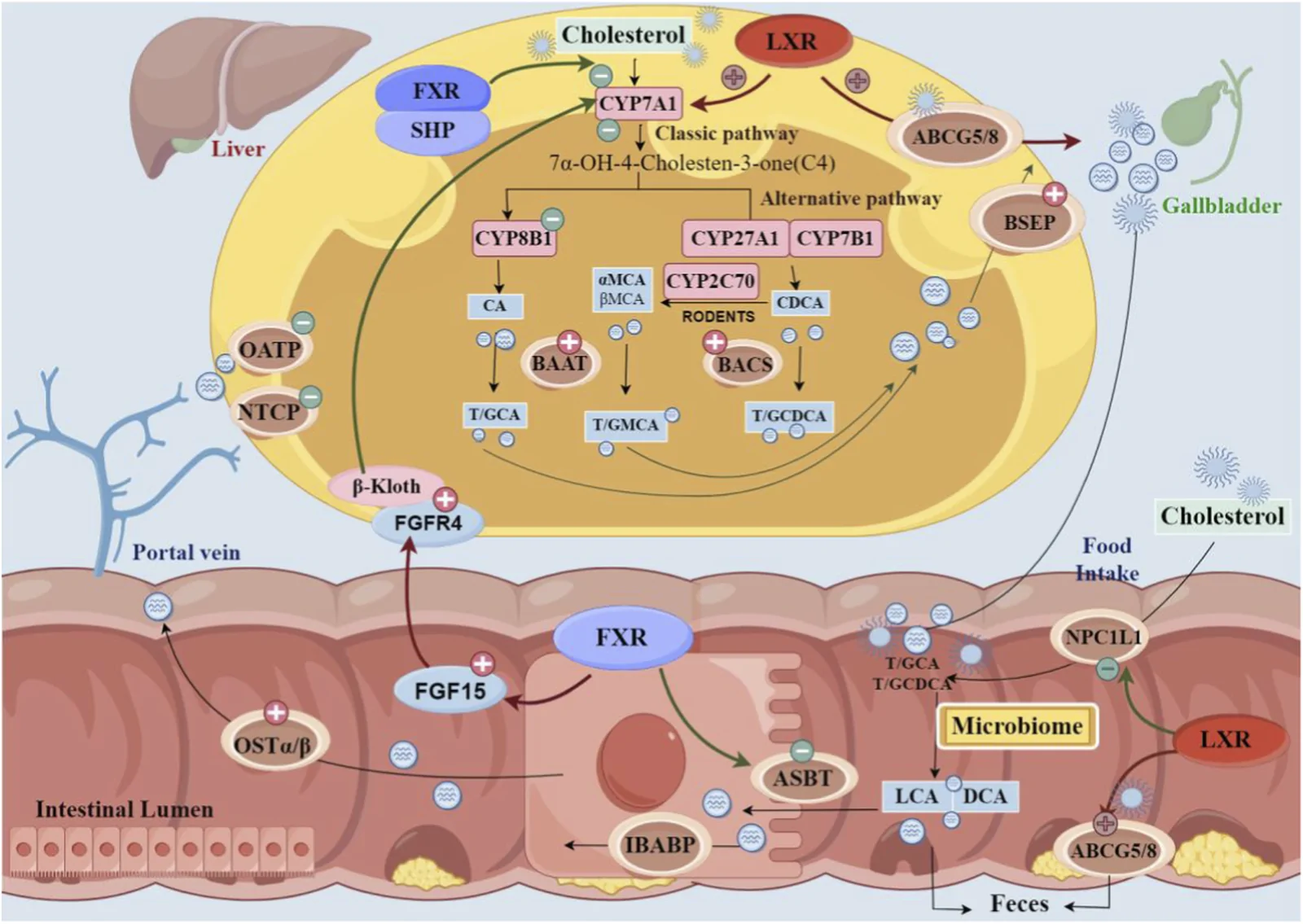

## Highlights

- DNE ameliorates cholesterol metabolism disorders in NAFLD mice *via* dua modulation of FXR/LXR.
- DNE modulates cholesterol absorption, biotransformation, and transport to attenuate NAFLD.
- DNE mediates the hypolipidemic activity of *Dendrobium nobile* Lindl., indicating its therapeutic potential against NAFLD.

## 1 Introduction

Non-alcoholic fatty liver disease (NAFLD) is characterized by excessive fat accumulation in hepatocytes (Ro et al., 2022). Globally, the prevalence of NAFLD is estimated to be approximately 25% of the population, with projections indicating a potential increase to 33.5% by 2030, imposing a significant economic burden on healthcare systems worldwide (Lin et al., 2024; Younossi et al., 2019). Dyslipidemia is a common complication observed in NAFLD patients, with hypercholesterolemia being the most prevalent manifestation (Sinha et al., 2019). Cholesterol, an essential functional molecule, is involved in various physiological processes, such as cell membrane formation and hormone synthesis. However, its high level has been linked to the development and progression of NAFLD (LI et al., 2021; Deprince et al., 2020). Therefore, maintaining cholesterol homeostasis is recognized as a crucial therapeutic strategy for NAFLD treatment.

*FXR* and *LXR* play pivotal roles in maintaining cholesterol homeostasis and are widely regarded as promising therapeutic targets for the treatment of NAFLD, especially in cases involving elevated cholesterol levels (Bao et al., 2022). Recent comprehensive reviews have further consolidated our understanding of NAFLD pathogenesis and emerging therapeutic strategies, highlighting the critical roles of bile acid signaling and nuclear receptor modulation in disease progression (Abo Nahas et al., 2026)**.** The conversion of cholesterol into bile acids (BAs) within the liver represents the principal pathway for cholesterol biotransformation. Cholesterol 7α-hydroxylase (*CYP7A1*) catalyzes the synthesis of primary BAs in liver cells (Yu Cai Lim et al., 2024), and its expression is predominantly regulated by *FXR* and *LXR* (Hiebl et al., 2018; Parlati et al., 2021). Research has demonstrated that *FXR* modulates *CYP7A1* expression through either *FXR-SHP* axis (Goodwin et al., 2000) or *FXR-FGF15/19* axis (Inagaki et al., 2005) in both the liver and intestine. *LXR* directly upregulates *CYP7A1* expression to promote the conversion of cholesterol to BAs in the liver. Additionally, through the regulation of the downstream genes, *FXR* facilitates BAs transport from the liver to the gallbladder, promotes its efflux, and diminishes BAs reabsorption in intestinal epithelial cells (WANG et al.; Li and chiang, 2014; Chiang and ferrell, 2020; WANG and Tontonoz, 2018).

*LXR* also augments the expression of ATP Binding Cassette Subfamily G Member5/8 (*ABCG5/8*) complex to enhance cholesterol excretion into the gallbladder. In the intestine, *LXR* promotes RCT expression, downregulates *NPC1L1*(Niemann-Pick C1-Like 1) expression to decrease intestinal cholesterol absorption, and elevates the expression of the intestinal *ABCG5/8* complex to enhance cholesterol excretion into the intestinal lumen, ultimately leading to a reduction in cholesterol accumulation in the body (Bradley et al., 2007; Duval et al., 2006; Yasuda et al., 2010; Dixon et al., 2021).

*Dendrobium nobile* Lindl. (*D. nobile*) is a renowned traditional Chinese medicine with diverse therapeutic properties, including nourishing the stomach, replenishing Yin, fortifying the liver and kidneys, and strengthening muscles and bones (Bao et al., 2022). In clinical practice, *D. nobile* is employed to treat a range of digestive disorders and metabolic diseases, particularly those related to the liver. Contemporary research has revealed that the primary active constituents of *D. nobile* encompass alkaloids (Liu et al., 2023a; Li et al., 2023). The alkaloids demonstrated the ability to effectively lower total cholesterol and triglyceride levels in both the serum and liver of mice with hypercholesterolemia resulting from a high-fat diet (Huang et al., 2019). Additionally, studies have demonstrated that *D*. *nobile* Lindl. significantly improves glucose and lipid metabolism disorders in NAFLD mouse models fed a high-fat diet, with further validation through *in vitro* cellular experiments (Xu et al., 2024; Sun et al., 2025). In addition, *D*. *nobile* Lindl, has also been documented to effectively ameliorate streptozotocin (STZ)-induced elevation of blood glucose and lipid levels to protect against STZ-induced fatty liver degeneration (Huang et al., 2013). Overall, the alkaloids exhibit promising hepatoprotective properties.

DNE is the prominent and defining component of *D*. *nobile* Lindl (Liu et al., 2023b), standing out as the most abundant alkaloid found within this plant species (LUO et al., 2024). Reports have demonstrated that DNE can significantly decrease liver and blood lipid levels in mice modeling NAFLD (XU et al., 2024). It influences the fatty acid oxidation process by upregulating the expression of Peroxisome Proliferator Activated Receptor Alpha (PPARα) and its downstream fatty acid metabolism-related genes, thereby reversing the progression of NAFLD. Consequently, from the perspective of cholesterol metabolism, it is crucial to further investigate the molecular mechanisms underlying DNE’s effects on NAFLD, in order to elucidate the material basis and mechanism of the lipid-lowering effect of *D. nobile*. Unlike our previous work on PPARα-mediated fatty acid oxidation, this study specifically addresses cholesterol-bile acid homeostasis *via* FXR/LXR pathways.

## 2 Materials and methods

### 2.1 Materials

DNE was purchased from Chengdu Pusi Bio-technology Co Ltd (Cat. No. PS2179-0020). Cholesterol (C3045) were obtained from Merck Life Sciences Co, Ltd. (Darmstadt, Germany). Chenodeoxycholic acid (HY-76847), (Z)-Guggulsterone (HY-110066), GW3965 (HY-10627A), SR9243 (HY-16972) were purchased from MedChemExpress (USA). Total Cholesterol (TC) assay kit (Cat. A110-1-1), Triglyceride (TG) assay kit (Cat. A111-1-1), Low Density cholesterol lipoprotein (LDL-C) assay kit (Cat. A112-1-1), High Density cholesterol lipoprotein (HDL-C)assay kit (Cat. A113-1-1) were obtained from Nanjing Jiancheng Bioengineering Institute (Nanjing, China).42 kinds of bile acid standards were purchased from Zhi Bo Biotech Products Co., Ltd (Shanghai, China). Simvastatin was obtained from Xinqi Pharmaceutical Co., Ltd (Shandong, China).

### 2.2 Animals and experimental protocol

C57BL/6 male mice, weighing 18 ± 2 g, were purchased from Hunan Slack Jingda Laboratory Animal Co, Ltd. (license number: SCXK2019-0004). These mice were housed in the animal center of Zunyi Medical University, where they were maintained under controlled conditions with an ambient temperature ranging from 21 °C to 25 °C, humidity levels between 56% and 65%, and provided with unrestricted access to food and water. Additionally, they were subjected to a 12/12 h balanced light-dark cycle. All animal procedures were conducted in compliance with the NIH Guide for the Care and Use of Laboratory Animals (eighth edition) and the ARRIVE guidelines 2.0, and were approved by the Animal Experiment Ethics Committee of Zunyi Medical University ([2021]2-606). After a 1-week acclimatization period, the mice were randomly assigned to either the normal diet group (ND) or the high-fat diet group (HFD) and provided with regular maintenance feed (10% fat, Jiangsu Synergy, #1010097) or high-fat feed (Jiangsu Synergy, XTHF60, which provided 20% of energy from protein, 60% from fat, and 20% from carbohydrates), respectively (XU et al., 2024). The mice with successful modeling were randomly divided into five groups, including high-fat diet group (HFD), low-dose DNE group (DNL, 5 mg/kg/day), medium-dose DNE group (DNM, 10 mg/kg/day), high-dose DNE group (DNH, 15 mg/kg/day) and positive drug simvastatin group (SIV, 20 mg/kg/day), with five mice in each group, and they continued to be fed with high-fat diet; the normal control group (ND), with five mice, continued to be fed with normal maintenance diet. DNE and simvastatin were prepared into suspensions of with 0.4% sodium carboxymethylcellulose (CMC-Na) solution, and the drugs were administered at a ratio of 0.1 mL of drug solution to 20 g of body weight. The mice in the ND group and HFD group were given corresponding volumes of 0.4% CMC-Na solution by gavage once a day for 17 weeks. The weight, hair, and mental state of the mice were observed and recorded every day.

Mice should take 9-18 mg/kg of DNE per day; In the previous study results of our group (Li et al., 2023; Liu, 2020), 15 mg/kg/day of Total alkaloid of *Dendrobium nobile* Lindl (DNLA) had the best lipid-lowering effect, and hepatotoxicity occurred when the dose reached 60 mg/kg/day. In summary, 5/10/15 mg/kg of DNE was used for follow-up experiments. Liver imaging changes were assessed by B-ultrasound using the Small Animal High Resolution Imaging System (Visual Sonics, Canada). At the end of the experiment, mice were deeply anesthetized with isoflurane using a small animal anesthesia machine (Rayward, China). After blood collection from the eyeball, the deeply anesthetized mice were humanely euthanized by cervical dislocation, performed by trained personnel in accordance with the AVMA Guidelines for the Euthanasia of Animals (2020 edition). Successful euthanasia was confirmed by respiratory arrest and lack of pedal reflexes, and liver tissue sections were obtained for H&E staining to observe pathological changes. The remaining liver tissue, gall bladder, ileum and ileum contents were promptly placed in liquid nitrogen and then transferred to an ultra low temperature freezer at −80 °C for future study.

### 2.3 Cell culture

Human hepatocellular carcinoma HepG2 cells were procured from Procell Life Sciences Ltd (Wuhan, China) and cultured in high-glucose DMEM medium, supplemented with 10% fetal bovine serum (FBS) and 1% double antibody. The cells were incubated at 37 °C in a 5% CO_2_ atmosphere and allowed to grow until they reached 80%–100% confluence.

The cells were inoculated into a six-well plate at a density of 1.5 X 10^5^ cells/mL and incubated with high-glucose DMEM complete medium supplemented with cholesterol for 24 h. Subsequently, cells were treated with varying concentrations of DNE (0.001, 0.01, 0.1, 1, 10, 100 μM), CDCA, GS, GW or SR for 24 h.

### 2.4 Biochemical indicators detection

About 10 mg of liver tissue was weighed on ice quickly, mixed with chilled absolute ethanol at a ratio of 1:9, ground, then centrifuged at 12,000 rpm, 4 °C, 15 min and the supernatant was taken. TC, TG, HDL-C, LDL-C levels in the serum and liver supernatant were measured according to the instructions.

### 2.5 Liver histological analysis

Hepatic tissue was fixed in a 10% neutral methanal solution for 48 h. After progressive dehydration in different concentrations of ethanol, it was soaked in xylene for 3 h and embedded in 5-µm-thick paraffin slices aft different concentrations being immersed in paraffin. The paraffin slices were baked at 60 °C for 40 min and then soaked in xylene twice for 10 min each time. Subsequently, the slices were subjected to dewaxing in anhydrous ethanol and 80% ethanol in turn, and then stained with hematoxylin for 15 min. After being washed with water for 30 s, the slices were stained with eosin for 2 min and then washed with water for 10 min. The slices were dehydrated twice and soaked in xylene for 5 min before being taken out and sealed with neutral resin for observation under an upright microscope.

### 2.6 B-ultrasound imaging monitoring

Mice that have been fasted overnight and after successful anesthesia by 2% isoflurane inhalation, maintained by continuous inhalation of 1% isoflurane in a mask. The cross-sectional image was intercepted, and the acquisition areas of the liver and kidney (avoiding blood vessels and bile ducts) were selected in this image area, and the average gray value of each region was recorded to calculate the echo ratio of the liver and kidney.

### 2.7 Real-time RT-qPCR

Total RNA was extracted from liver, ileum, and HepG2 cells using TRIzol according to the manufacturer’s instructions. cDNA was synthesized from the purified RNA through reverse transcription. Real-time PCR was performed using a SYBR Green polymerase reaction kit (MedChemExpress, NJ, USA) with target gene-specific primers on a real-time quantitative PCR instrument (BIO-RED, USA). The amplification protocol included pre-denaturation at 95 °C for 5 min, followed by 40 cycles of denaturation at 95 °C for 30 s and annealing/extension at 55 °C for 30 s. Melting curve analysis was automatically generated by the instrument’s temperature programming system (El behery et al., 2025).

### 2.8 Western blot

Total protein was extracted from liver tissues and quantified using the BCA method. Equal amounts of protein samples (30 μg/lane) were denatured in a 100 °C metal bath, separated by SDS-PAGE, and subsequently transferred onto PVDF membranes. After blocking with 5% skim milk for 1 h, the membranes were washed with TBST and incubated with primary antibodies against NR1H3 (Cat. 14351-1-AP, Proteintech), NR1H4 (Cat. 25055-1-AP, Proteintech), GAPDH (Cat. 10494-1-AP, Proteintech) and CYP7A1 (Cat. 18054-1-AP, Proteintech) at 4 °C for 12-16 h. Following three washes with TBST, the membranes were incubated with an HRP-conjugated AffiniPure Goat Anti-Rabbit IgG (H + L) secondary antibody (Cat. SA00001-2, Proteintech) at room temperature for 2 h. After three additional TBST washes, protein bands were visualized using a chemiluminescence Western blot detection kit, and the band intensities were quantified using Image Lab software (version 1.8.0) (Alzahrani et al., 2024a).

### 2.9 Changes in bile acids examined by LC-MS/MS

Bile acid metabolomics was performed based on the literatures of our group (YE et al., 2023; Lu et al., 2018). Approximately 30 mg of liver tissues was homogenized in 90 μL of acetonitrile on ice, 2 μL bile was diluted in 98 μL chilled sterile water, 30 mg of ileum contents was homogenized in 300 μL of acetonitrile on ice. The homogenates were centrifuged at 14,300 rpm and 4 °C for 10 min. An aliquot of 250 μL of supernatants was evaporated by nitrogen gas blow and dissolved in 50% of methanol (100 μL). After centrifugation at 14,300 rpm and 4 °C for 10 min, the supernatants were used for quantification.

Bile acids was determined on API 4000TM using a Hypersil GOLD C18 column (2.1 150 mm, 1.9 μm) as the stationary phase and the mobile phase consisting of 0.1% formic acid, water (A), and acetonitrile (B) was eluted gradually. The injection volume was 2 μL, the flow rate was 0.5 mL/min, and the column temperature was 30 °C.Mass spectrometry was performed using an electrospray ionization source (ESI) with a positive ion multiple reaction detection mode (MRM) (Alzahrani et al., 2024b). The ESI-MS/MS operating parameters used in this method are set as follows: ion spray voltage −4500 V, curtain gas 25 psi, ion source gas 140 psi, ion source gas 245 psi, ion transfer tube temperature 450 °C, collision gas 8.

### 2.10 Statistical method

The experimental data were expressed as mean ± *Standard error (SEM)*. One-way analysis of variance (ANOVA) was conducted using SPSS 29.0 statistical software. For homogenous variances, the least significant difference (LSD) method was employed, while the Dunnett’s T3 method was used for cases with non-homogeneous variances. A significance level of *P* < 0.05 was considered statistically significant. Given the hypothesis-driven nature of our analyses with predefined comparisons (e.g., HFD vs. DNE groups) and focused targets (FXR/LXR-related genes and specific BA species), no correction for multiple testing was applied.

## 3 Results

### 3.1 Effect of DNE on ameliorating lipid metabolism disorders in NAFLD mice

To assess the effects of DNE on hepatic steatosis and morphology, we performed H&E staining on liver tissues Figure 1A. ND (normal diet), HFD (high-fat diet providing 60% of energy from fat, fed for 12 weeks to establish NAFLD and continued during the 17-week treatment period), DNL (low-dose DNE, 5 mg/kg/day), DNM (medium-dose DNE, 10 mg/kg/day), DNH (high-dose DNE, 15 mg/kg/day), and SIV (simvastatin, 20 mg/kg/day). As illustrated, ND group mice exhibited intact liver lobules with neatly arranged hepatocytes and round, clear nuclei. In contrast, HFD group mice showed disordered hepatocyte architecture, marked swelling, nuclear shrinkage, diffuse fatty vacuoles, ballooning degeneration, and mild inflammatory infiltration. Compared with the HFD group, hepatic fat accumulation and steatosis were improved to varying degrees in the DNM, DNH, and SIV groups, suggesting that medium- and high-dose DNE reversed high-fat diet-induced hepatic morphological damage.

**FIGURE 1:**
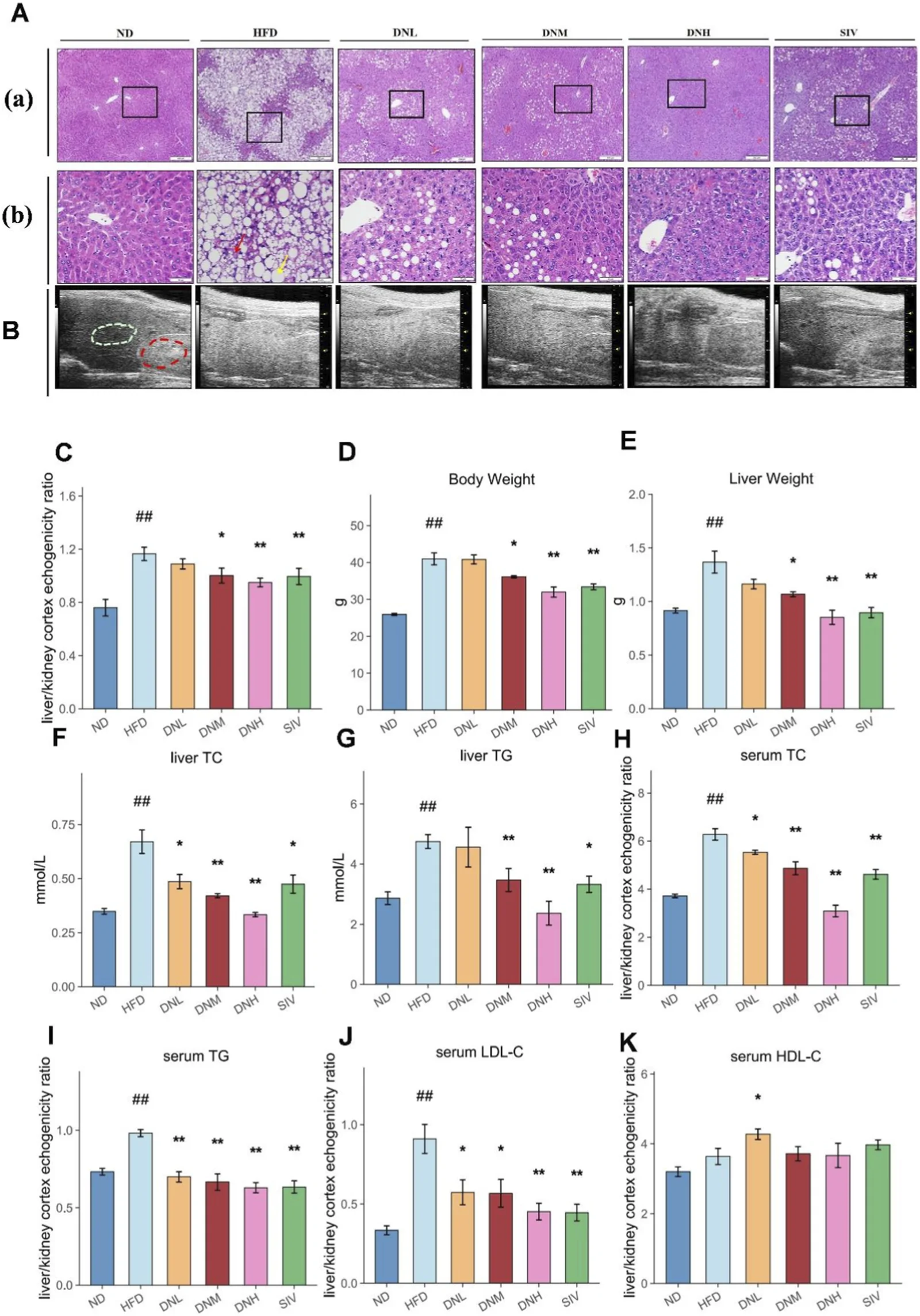
Effect of DNE on ameliorating lipid accumulation in NAFLD mice (n = 5). ND, normal diet; HFD, high-fat diet; DNL, low-dose DNE (5 mg/kg/day); DNM, medium-dose DNE (10 mg/kg/day); DNH, high-dose DNE (15 mg/kg/day); SIV, simvastatin (20 mg/kg/day). **(A)** Liver H&E staining. **(B)** Representative B-ultrasound images of liver and kidney. **(C)** Liver-to-kidney echo ratio. **(D–K)** Serum and hepatic biochemical parameters. Data are mean ± *SEM*. ##*P* < 0.01 vs. ND; *P < 0.05, **P < 0.01 vs. HFD.

To evaluate liver fat deposition *in vivo*, we conducted abdominal ultrasound imaging. As depicted in Figures 1B,C, compared with the ND group, HFD mice exhibited punctate hyperechogenicity, mild to moderate hepatomegaly, and rounded liver margins, along with a significantly increased liver-to-kidney echo ratio, indicating substantial hepatic fat accumulation. This ratio was markedly reduced in the DNM, DNH, and SIV groups compared to the HFD group, suggesting that medium- and high-dose DNE or simvastatin administration alleviated hepatic lipid deposition.

To further determine the impact of DNE on systemic and hepatic lipid profiles, we measured body weight, liver weight, and lipid levels. As shown in Figures 1D–J, compared with the ND group, the HFD group showed significant increases in body weight, liver weight, hepatic TC and TG, as well as serum TC, TG, and LDL-C levels. These elevations were significantly attenuated in the DNM, DNH, and SIV groups. In summary, medium- and high-dose DNE administration effectively reduced lipid accumulation in both serum and liver and improved lipid metabolism disorders in NAFLD mice.

### 3.2 Effect of DNE on the level and profiles of BAs in NAFLD mice

LC-MS/MS was used to detect 42 bile acid species within 46 min. The total ion chromatogram (TIC) showed that each peak exhibited good resolution and shape. The chromatographic peaks were identified by standard substances, and 26 kinds of BAs were detected in the liver Figure 2A, 28 kinds of BAs were detected in the bile Figure 2B, 31 kinds of BAs were detected in the ileum contents Figure 2C. The chromatographic peak area obtained was converted into the concentration of BA by internal standard method.

**FIGURE 2:**
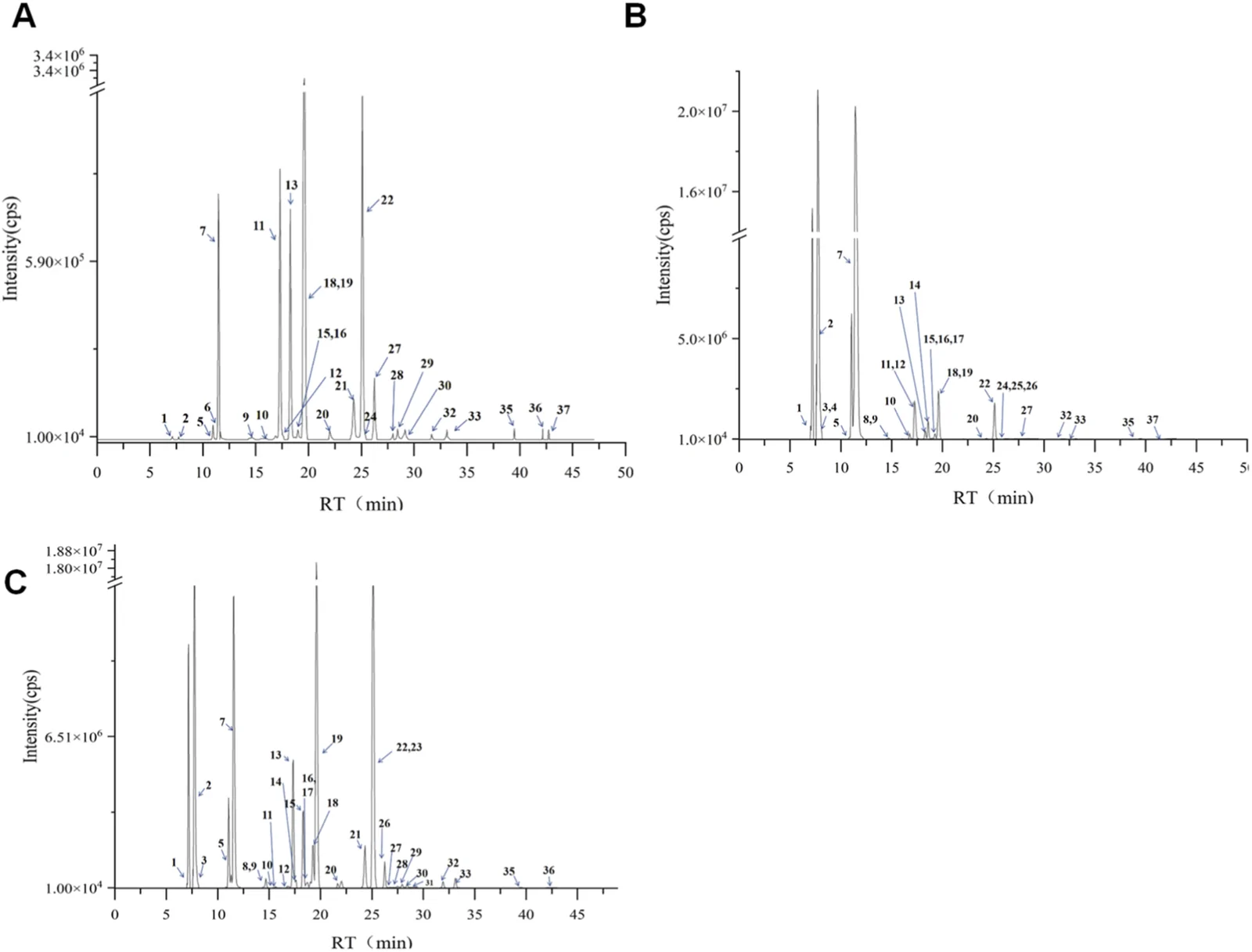
TIC of bile acids in liver, bile and ileum contents detected by LC-MS/MS. **(A)** TIC of bile acids in liver detected by LC-MS/MS. **(B)** TIC of bile acids in bile detected by LC-MS/MS. **(C)** TIC of bile acids in ileum contents detected by LC-MS/MS.

Tip: 1. TαMCA; 2. TβMCA; 3. THCA-3S; 4. TCDCA-3G; 5. TUDCA; 6. GDHCA; 7. TCA; 8. GHCA-3S; 9. UCA; 10. 7,12-D-LCA; 11. TCDCA; 12. GCA; 13. βCA; 14. GUDCA; 15. αMCA; 16. TDCA; 17. NorCA; 18. 7-DCA; 19. βMCA; 20. γMCA; 21. βUDCA; 22. CA; 23. 3βHDCA; 24. TLCA; 25. HDCA; 26. GDCA; 27. 6-keto-LCA; 28. 7-keto-LCA; 29. NorDCA; 30. 12-keto-LCA; 31. ApoCA; 32. CDCA; 33. DCA; 34. ILCA; 35. IDCA; 36. LCA; 37. alloLCA.

Compared with the ND group, the level of total BAs in liver, bile, ileum contents of the mice in HFD group were significantly reduced Figure 3A; Compared with the HFD group, the level of total BAs were significantly increased after DNE administration. These results suggest that DNE may promote the conversion of cholesterol to BAs and increase the level of bile acids.

**FIGURE 3:**
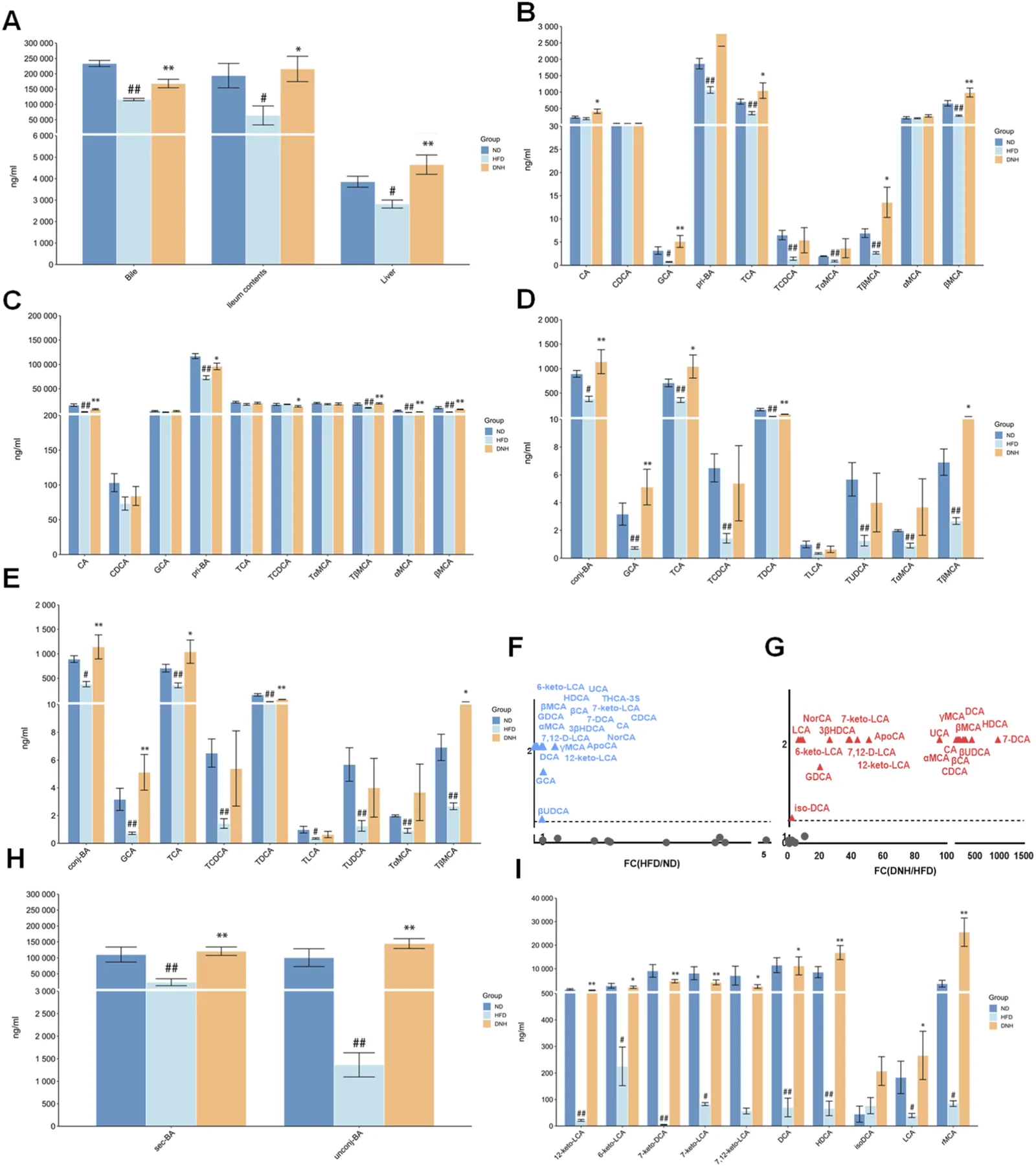
Effect of DNE administration on the level and profiles of BAs in NAFLD mice (n = 5). Bile acid profiling was performed using LC-MS/MS to quantify 42 bile acid species in liver tissues, bile, and ileum contents. **(A)** Total BA levels in liver, bile, and ileum contents. **(B)** Primary BA levels in liver. **(C)** Primary BA levels in bile. **(D)** Conjugated BA levels in liver. **(E)** Conjugated BA levels in bile. **(F)** Differential BAs in ileum contents: ND vs. HFD. **(G)** Differential BAs in ileum contents: HFD vs. DNH. **(H)** Secondary and unconjugated BA levels in ileum contents. **(I)** DCA and LCA levels in ileum contents. Abbreviations: ND, normal diet; HFD, high-fat diet; DNH, DNE high-dose (15 mg/kg/day). Data are mean ± *SEM*. #*P* < 0.05, ##*P* < 0.01 vs. ND; *P < 0.05, **P < 0.01 vs. HFD.

There are significant changes in the level of primary bile acids (pri-BA, include CA, TCA, GCA, CDCA, TCDCA, αMCA, βMCA, TαMCA, TβMCA) in the liver Figure 3B and bile Figure 3C. Compared with the ND group, the level of pri-BA in the liver and bile of the HFD group mice were significantly reduced, while significantly increased after DNE administration, suggesting that DNE administration may promote the synthesis of BAs. At the same time, there are also substantial changes in the level of conjugated bile acids (conj-BA, include GCA, TβMCA, TCA, TCDCA, GCDCA, TLCA, TUDCA, TDCA, TαMCA, GDCA, GUDCA, GHCA-3S, TCDCA-3G, THCA-3S) in the liver Figure 3D and bile Figure 3E. Compared with the ND group, the level of conj-BA in the liver and bile of the HFD group mice was reduced, while significantly increased after the administration of DNE, suggesting that the administration of DNE may promote the binding of BAs to amino acids, increase the hydrophilicity of BAs, and facilitate the excretion into the gallbladder for storage.

Besides, high-fat diet Figure 3F,DNE Figure 3G administration significantly transformed the BA profiles in the ileum contents of mice, and further analysis of differential bile acids found that the level of secondary bile acids (sec-BA) and unconjugated bile acids (unconj-BA) were significantly changed. Compared with the ND group, the levels of sec-BA and unconj-BA in the ileum contents of the HFD group mice were significantly decreased, and compared with the mice in the HFD group, these BAs significantly increased after DNE administration Figure 3H. And the level of the secondary metabolites of DCA and LCA Figure 3I were also significantly increased, suggesting that DNE administration may promote the conversion of BAs and enhance the metabolism of BAs in the intestine.

### 3.3 Effect of DNE on the synthesis of BAs in NAFLD mice

RT-qPCR was used to examine the expression level of target genes related to BA synthesis. As shown in Figure 4A, compared with the ND group, the expression levels of *Cyp7a1*, *Cyp8b1* and *Cyp7b1* in the liver of the HFD group mice decreased significantly, while the expression levels of *Cyp7a1*, *Cyp8b1*, *Cyp27a1* and *Cyp7b1* were significantly upregulated after DNE administration. That is, the mRNA expression level of BA synthesis-related genes was significantly upregulated after DNE administration, promoted the synthesis of BA, which was consistent with the changes in level and profiles of BAs. Compared with the ND group, the expression levels of the nuclear receptor *Fxr*, which is crucial for regulating BA synthesis, and its partners *Shp*, *Lxrα* and *Lxrβ* in the liver of mice in the HFD group were significantly decreased, and significantly upregulated after DNE administration.

**FIGURE 4:**
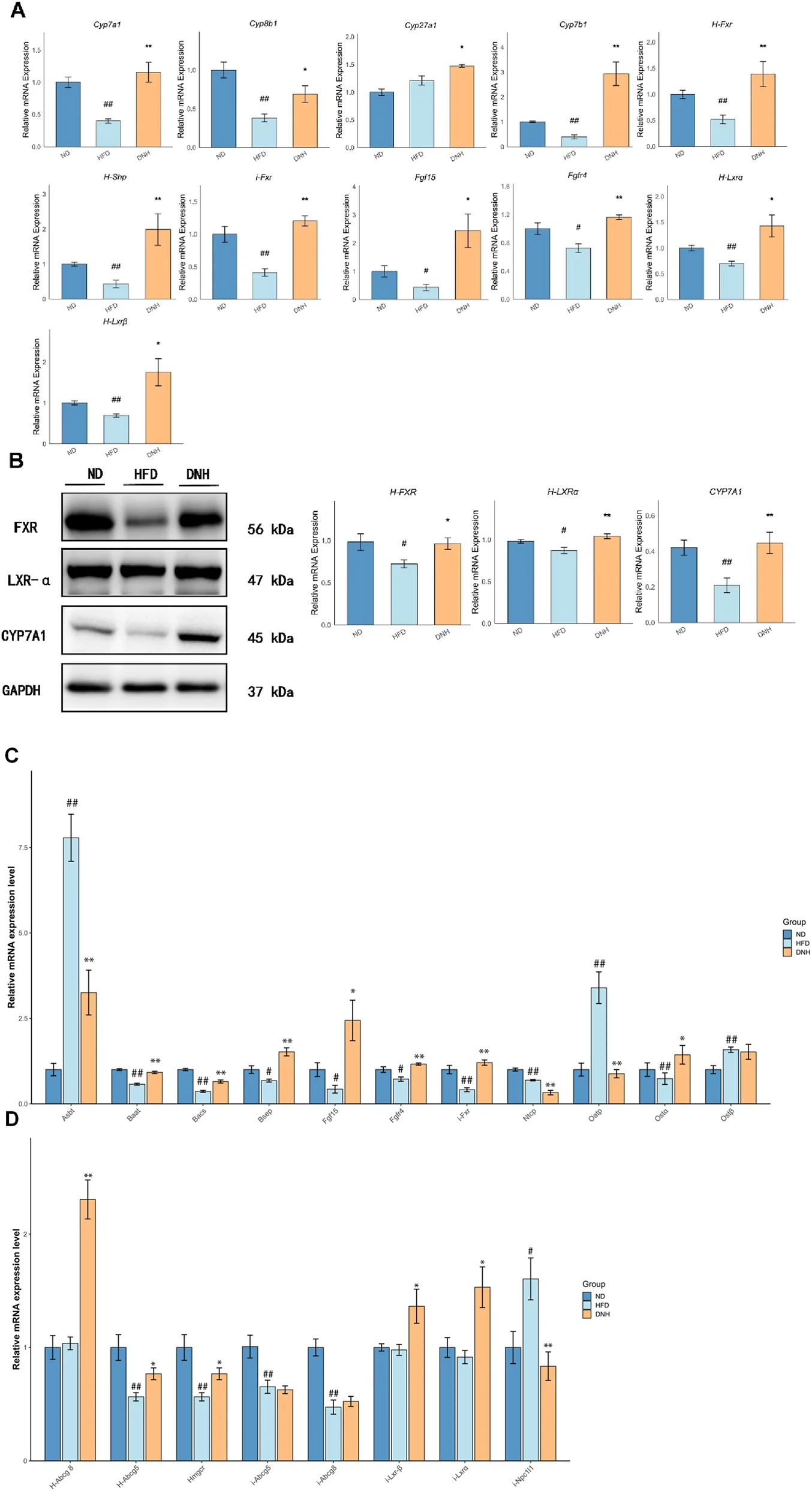
Effect of DNE Administration on *FXR* and *LXR* Genes Associated with BA Synthesis in NAFLD Mice (n = 5). ND, normal diet; HFD, high-fat diet; DNH, high-dose DNE (15 mg/kg/day). **(A)** RT-qPCR analysis of BA synthesis-related genes in the liver. **(B)** Western blot analysis of *FXR, LXRα,* and *CYP7A1* protein in the liver. **(C)** Expression of FXR and its downstream genes: liver (*Bacs, Baat, Bsep, Ntcp, Oatp, Fgfr4*) and ileum (*Fxr, Fgf15, Asbt, Ostα, Ostβ*). **(D)** Expression of LXR and its downstream genes: ileum (*Lxrα, Lxrβ, Npc1l1*) and liver (*Abcg5/8*). Data are mean ± *SEM*. #*P* < 0.05, ##*P* < 0.01 vs. ND; *P < 0.05, **P < 0.01 vs. HFD.

According to the WB results Figure 4B, compared with the ND group, the protein expression levels of FXR*,* LXRα and Cyp7a1 in the liver of HFD mice were significantly decreased, whereas they were significantly increased after DNE administration.

As shown in Figure 4C, DNE not only has a regulatory effect on the *Fxr*-*Shp* axis in the liver, but also has a significant effect on *Fxr-Fgf15* in the ileum. Compared with the ND group, the expression levels of *Fxr*, *Fgf 15* in ileum, as well as *Fgfr4* in liver of HFD group mice were significantly decreased, while significantly upregulated after administration of DNE. In the liver, DNE significantly altered the expression of downstream genes of *Fxr*. Compared with the ND group, the expression levels of *Bacs*, *Baat*, *Bsep* and *Ntcp* in HFD group mice decreased significantly, and *Oatp* increased significantly. Compared with the HFD group, the expression levels of *Bacs* and *Baat* in the DNH group mice increased significantly. DNE also significantly altered the expression levels of downstream genes of *Fxr* in the ileum. Compared with the ND group, the expression levels of *Asbt* and *Ostβ* in the HFD group mice increased significantly, and *Ostα* decreased significantly. Compared with the HFD group, the expression level of *Asbt* in the ileum in the DNH group decreased.

As can be learnt from Figure 4D, DNE also has a significant regulatory effect on the expression level of *Lxrs* in the ileum. Compared with the ND group, there was no significant change in the expression levels of *Lxrα* and *Lxrβ* in the ileum of the HFD group mice. Compared with the HFD group, the expression levels of *Lxrα* and *Lxrβ* were significantly upregulated after DNE administration. Furthermore, DNE significantly altered the expression of downstream genes of *Lxrs*. Compared with the ND group of mice, the transcription level of *Abcg5*, the cholesterol efflux transporter, was significantly downregulated in the HFD group mice liver, while the expression of *Abcg5/8* in the DNH group was significantly increased. Moreover, in the ileum, compared with the ND group, the expression level of *Npc1l1* in the HFD group mice, which control the absorption of cholesterol, was significantly increased, While DNE significantly decreased the expression of *Npc1l1*.

These findings indicate that the dual modulation of *Fxr* and *Lxr* signaling pathways by DNE promotes bile acid synthesis and cholesterol homeostasis, which contributes to the amelioration of metabolic disorders following high-fat diet.

### 3.4 *In vitro* validation and mechanism study of DNE on reducing cholesterol accumulation in HepG2 cells

Based on the combined results of cell viability and intracellular TC levels, co-culturing HepG2 cells with 40 μM cholesterol (CHOL) for 24 h was established as the condition for constructing a cholesterol-accumulating hepatocyte model (Figures 5A,B). To determine the appropriate DNE dosage, HepG2 cells in the cholesterol-loaded model were treated with DNE at concentrations ranging from 0.1 nM to 10 μM for 24 h. Cell viability assays showed that DNE at 1 nM, 10 nM, and 100 nM maintained high cell viability (>90%), whereas concentrations ≥1 μM exhibited significant cytotoxicity (Figure 5C). Intracellular TC measurements further demonstrated that these three concentrations effectively reduced cholesterol accumulation in a concentration-dependent manner, with 100 nM showing the most pronounced effect (Figure 5D). Therefore, DNE concentrations of 1 nM, 10 nM, and 100 nM were selected as the appropriate dosage range for subsequent experiments. Consistent with the results *in vivo* Figure 4A, 100 nM of DNE administration also significantly increased the expression level of *CYP7A1*, *CYP27A1*, *FXR*, *SHP* and *LXRα* Figures 5E–I. These results indicate that DNE directly reduces cholesterol accumulation in hepatocytes and continuously modulates the expression of key genes in the FXR/LXR pathway, consistent with its *in vivo* effects.

**FIGURE 5:**
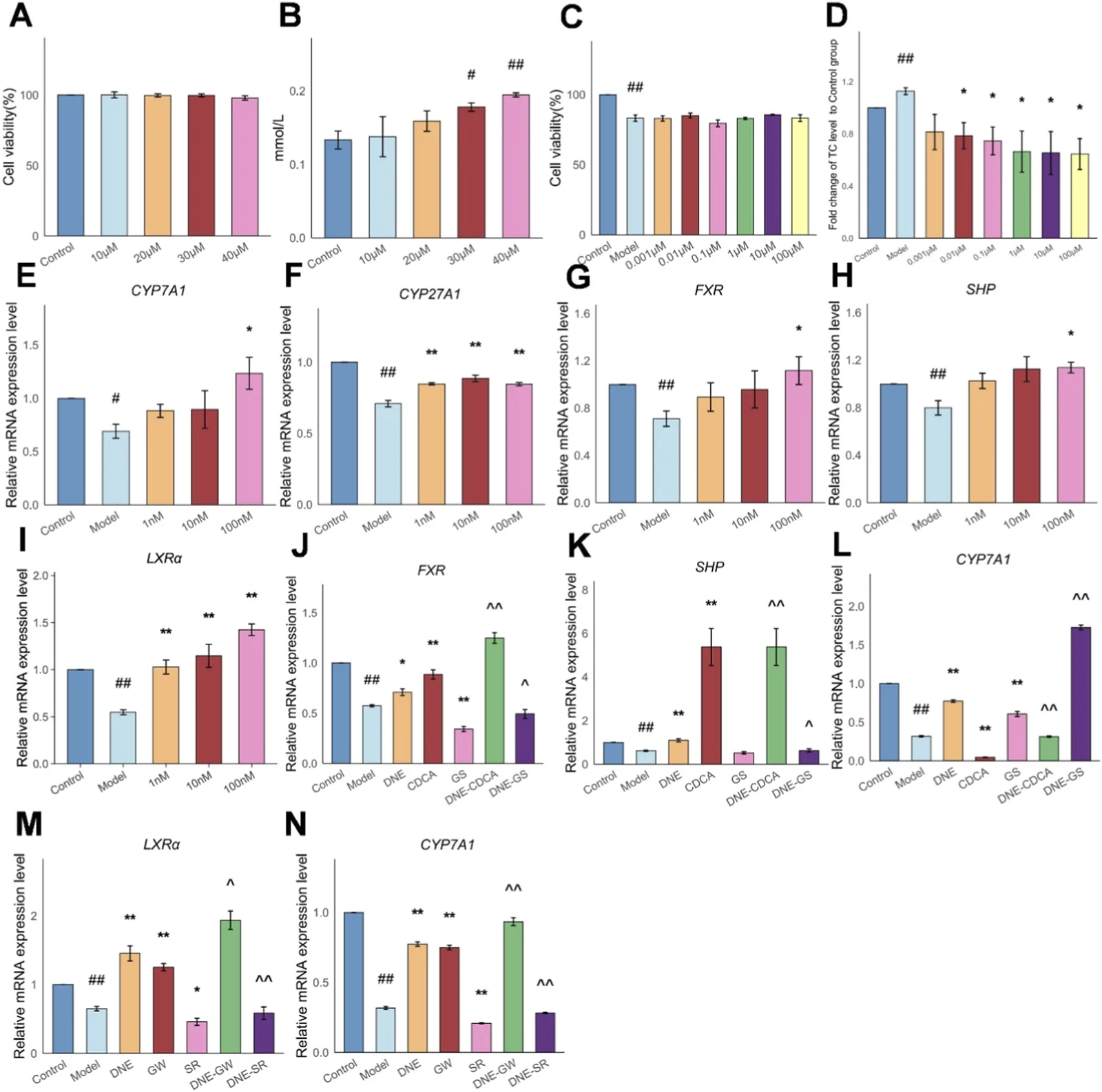
DNE reduces HepG2 cell cholesterol accumulation(n = 3). Cells were pretreated with 40 μM cholesterol (CHOL) for 24 h to establish a cholesterol-loaded hepatocyte model, followed by treatment with DNE (100 nM) alone or in combination with FXR agonist CDCA (50 μM), FXR antagonist GS (10 μM), LXR agonist GW3965 (GW, 5 μM), or LXR antagonist SR9243 (SR, 10 μM) for an additional 24 h. **(A)** Cell viability after 24 h co-culture with CHOL. **(B)** Intracellular TC levels after CHOL treatment. **(C)** Cell viability after DNE treatment. **(D)** Intracellular TC levels after DNE treatment. **(E–I)** mRNA expression of *CYP7A1*, *CYP27A1, FXR, SHP,* and *LXRα* after DNE treatment. **(J–L)** Transcriptional levels of *FXR*, *SHP*, and *CYP7A1* after co-treatment with DNE and FXR agonist (CDCA) or antagonist (GS). **(M,N)** Expression of *LXRα* and *CYP7A1* after co-treatment with DNE and LXR agonist (GW3965) or antagonist (SR9243). Abbreviations: Control (untreated), Model (CHOL alone), DNE (100 nM), CDCA (50 μM), GS (10 μM), GW (GW3965, 5 μM), SR (SR9243, 10 μM). Combination groups: DNE-CDCA, DNE-GS, DNE-GW, DNE-SR. Data are mean ± *SEM*. #*P* < 0.05, ##*P* < 0.01 vs. Control; *P < 0.05, **P < 0.01 vs. Model; ^*P* < 0.05, ^ ^*P* < 0.01 vs. DNE alone.

To further investigate the role of *FXR/LXR* in DNE’s improvement of cholesterol dysregulation, HepG2 cells were treated with the *FXR* agonist chenodeoxycholic acid (CDCA) or the *FXR* antagonist (Z)-Guggulsterone (GS). *LXR* agonist GW3965 (GW), and *LXR* antagonist SR9243 (SR) were administered to HepG2 cells. RT-qPCR was employed to assess mRNA expression levels of the *FXR/SHP/LXR/CYP7A1* genes.

As shown in Figures 5J,K, compared with the model group, both DNE and the FXR agonist CDCA significantly upregulated the expression of *FXR* and *SHP*, while the *FXR* inhibitor GS significantly downregulated *FXR* expression. Furthermore, compared to DNE alone, the combination of DNE and CDCA further enhanced *FXR* and *SHP* expression, whereas the combination with GS significantly suppressed both. Regarding *CYP7A1* Figure 5L, both DNE and GS significantly increased their expression levels, while CDCA significantly inhibited its expression. Notably, the induction of *CYP7A1* by DNE was attenuated when combined with CDCA but further enhanced when combined with GS. These results suggest that *FXR* activation antagonizes DNE’s upregulation of *CYP7A1*, while *FXR* inhibition synergistically enhances this effect, indicating that DNE may regulate *CYP7A1* expression in an *FXR-*dependent manner.

As shown in Figure 5M, both DNE and the *LXR* agonist GW3965 significantly upregulated *LXRα* expression, whereas the *LXR* inhibitor SR9243 suppressed it. Combination treatments revealed that GW3965 synergistically enhanced DNE’s effect on *LXRα* expression, while SR9243 markedly attenuated it. For *CYP7A1* expression Figure 5N, DNE and GW3965 individually promoted its expression, with their combination demonstrating synergistic upregulation. In contrast, SR9243 not only suppressed *CYP7A1* expression alone but also significantly counteracted DNE-induced upregulation when co-administered. These findings demonstrate that DNE regulates *CYP7A1* through the *LXRα* pathway: *LXRα* activation synergistically enhances whereas *LXRα* inhibition attenuates this regulatory effect.

In summary, these findings suggest that DNE upregulates *CYP7A1* expression through a mechanism involving the coordinated modulation of both *FXR* and *LXR* pathways. The bidirectional response to receptor-specific agonists and antagonists highlights the potential crosstalk between these nuclear receptors in mediating the cholesterol-lowering actions of DNE.

## 4 Discussion

While our earlier studies demonstrated DNE’s regulation of *PPARα-*mediated fatty acid oxidation, the present project investigates a distinct mechanism—whether DNE ameliorates cholesterol metabolism disorders through FXR/LXR pathways—using a high-fat diet-induced mouse model of NAFLD. The H&E staining and imaging results showed that the extent of lipid accumulation and steatosis in the liver of mice in the DNM group and DNH group was significantly reduced, while the levels of TC and LDL-C in the liver and serum were significantly decreased, indicating that DNE can improve the lipid accumulation caused by a high-fat diet, which is one of the material bases for the lipid-lowering effect of *D. nobile*. Of note, the lack of significant differences among tested doses for several metabolic parameters suggests that 10 mg/kg may already approach the maximal effective dose, possibly reflecting saturable target engagement. Unlike simvastatin, DNE did not restrict the expression of Hmgcr and thus inhibit *de novo* cholesterol synthesis, while LC-MS/MS systematically examined the changes in the level and profiles of BAs in the liver, bile, and ileum contents of mice, and found that DNE had a significant effect on the conversion of cholesterol. Compared with the HFD group, the levels of total BAs in the liver, bile, and ileum contents, and the primary bile acids in the liver and bile of the DNH group mice were significantly increased. At the same time, the levels of mRNA expression and protein expression of the rate-limiting enzyme Cyp7a1 in the classical pathway of bile acid synthesis increased significantly, suggesting that DNE improved the cholesterol metabolism disorders and reduced the accumulation of cholesterol in the liver caused by a high-fat diet at least in part by promoting the synthesis of BAs. Notably, the generation of secondary bile acids largely depends on gut microbiota-encoded 7α-dehydroxylase (e.g., the bai operon); therefore, the elevated secondary bile acid levels observed following DNE treatment may be partially mediated by alterations in gut microbiota composition or function. Although this possibility was not directly examined in the present study, future investigations incorporating metagenomics and germ-free animal models are warranted to elucidate the causal chain among DNE, gut microbiota, and bile acid metabolism.

In order to elucidate the mechanism by which DNE regulates BA synthesis, we next examined the expression of key nuclear receptors that regulate BA synthesis. According to the literatures (LU et al., 2000), the nuclear receptor FXR forms a heterodimer with the co-inhibitor Shp in the liver, which binds to the response element of the Cyp7a1 promoter and inhibits Cyp7a1 expression, which is one of the important pathways to regulate BA synthesis (INAGAKI et al., 2005; DAI et al., 2023; DASH et al., 2017). From the RT-qPCR results, the expression levels of FXR and Shp in the liver of mice were significantly upregulated after DNE administration, and the WB results also showed that the protein expression levels of FXR in the liver increased significantly after DNE administration. The upregulation of Cyp7a1 was contradictory to the upregulation of FXR and Shp in the liver, but was consistent with the significant change in Cholic acid (CA), a product of the classical pathway of BA synthesis in the liver. Since the intestinal FXR-FGF15/19 axis also plays an important regulatory role in Cyp7a1 (SHIN and OSBORNE, 2009; AL-AQIL et al., 2018), we speculate that it may be that the intestinal FXR exerts the induction of CYP7A1 by DNE. However, contrary to expectations, compared with the HFD group, DNE administration significantly upregulated the expression levels of FXR, Fgf15 and its receptor Fgfr4 in the intestine and liver of mice, that is, the FXR-FGF15/19 pathway in the gut was also activated. The phenomenon that expression levels of FXR and Cyp7a1 were significantly upregulated at the same time has also have been reported (LEE et al., 2021; XUE et al., 2024; Liu et al., 2023b). Therefore, we put forward another hypothesis, that is, in addition to FXR, DNE can also regulate Cyp7a1 through other pathways to promote BA synthesis.

In addition to the classical pathway, the expression levels of the key enzymes *Cyp27a1* and *Cyp7b1* in the alternative BA synthesis pathway were also significantly increased. *Cyp27a1* is a multifunctional cytochrome P450 enzyme that is mainly found in the mitochondria of cells and hydroxylates cholesterol at the 27th carbon atom to generate 27-hydroxycholesterol (27-HC) (ALFAQIH et al., 2017). 27-HC is a high-affinity endogenous ligand for *LXRα*, which activates *LXRα* directly and then upregulates the expression of *Cyp7a1* in the liver to promote the synthesis of BAs (ZHOU and HYLEMON, 2014). *Cyp7b1* further hydroxylates 25-HC, 26-HC, and 27-HC to reduce the hepatic toxicity of these oxysterols (BIASI et al., 2022). From the results of RT-qPCR and WB, the levels of transcription expression and protein expression of *LXR*α in mouse liver were indeed significantly upregulated after DNE administration, indicating that DNE may have induced the expression of *Cyp7a1* through hepatic *LXR*α.

Accumulating evidence suggests that dual modulation of *FXR* and *LXR* is critical in the development and progression of NAFLD (WANG and TONTONOZ, 2018; BAO et al., 2022; CALKIN and TONTONOZ, 2012; FAN et al., 2021). For instance, Acanthoic acid (AA)—a terpenoid isolated from *Acanthopanax senticosus*—exhibits anti-inflammatory and hepatoprotective effects. After 12 weeks of administration of a modified Lieber-DeCarli diet combined with AA, C57BL/6 mice in the AA-treated group showed significantly decreased body weight, liver index, serum triglycerides, and aminotransferase levels, while the expression of FXR and LXR in the liver was significantly upregulated, and the downstream AMPK-LKB1 and SIRT1 signaling pathways were activated; In AML12 cells stimulated with palmitic acid, the ability of AA to ameliorate lipid accumulation was greatly reduced after silencing of *FXR* or *LXR*, suggesting that AA-mediated dual activation of *FXR* and *LXR* represents an effective strategy to alleviate NAFLD progression (HAN et al., 2019). In a model induced by a Western diet with sugar water (WDSW), Withaferin A effectively reduced body weight, blood glucose, TC, and TG levels, decreased the release of *Tnf-α, IL-6, IL*-β and MCP-1, and ameliorated liver inflammation and fibrosis in diet-induced NAFLD mice. Consistent with vivo findings, Withaferin A reduced lipid accumulation in HepG2 and Huh7 cells induced by palmitate and oleate. This improvement may be attributed to the synergistic inhibition of NF-κB and TGF-β expression and release following dual activation of *FXR* and *LXR*, thereby mitigating liver injury induced by lipid accumulation (SHIRAGANNAVAR et al., 2023). Bin Dong et al. (DONG et al., 2019) found that obeticholic acid (a classical *FXR* agonist) did not increase the expression of Scavenger receptor class B type I (SR-BI) in hamsters on a normal diet. They demonstrated that upregulation of SR-BI expression required synergistic binding of *FXR* and *LXR* to recognition sequences within the intronic region of the SR-BI gene, as shown by *in vitro* DNA binding and luciferase reporter assays. These findings were further validated in cell models. YuChen Jiang et al. (JIANG et al., 2024). found that in a thioacetamide (TAA)-induced liver fibrosis model in C57BL/6 mice and in hepatic stellate cells (HSCs) stimulated with *TGF-β* or conditioned medium, activation of *FXR* and *LXR* was crucial for the interaction between HSCs and macrophages. This effect was mediated by inhibition of autophagy *via* sesame extracts (SEM). Silencing *FXR* or *LXR* led to increased expression of extracellular matrix (ECM)-related markers, inflammatory cytokines, and autophagy, thereby attenuating the anti-fibrotic effect of SEM. Collectively, these studies indicate that dual activation of *FXR* and *LXR* exerts synergistic beneficial effects in regulating energy metabolism, inflammation, fibrosis, and other processes relevant to NAFLD pathogenesis. Compared with these compounds, DNE exhibits distinct characteristics: unlike withaferin A, which acts *via* direct receptor binding, DNE modulates FXR/LXR signaling at the transcriptional level without requiring direct receptor interaction; unlike AA, which primarily exerts anti-inflammatory effects, DNE systematically regulates the entire cholesterol absorption-biotransformation-transport axis; and unlike obeticholic acid or sesame extracts, which target FXR or LXR predominantly in the liver, DNE coordinates both pathways across the liver-bile-ileum axis. These distinctions highlight DNE as a multi-target modulator of cholesterol-bile acid homeostasis.

In addition to its role in BA synthesis, the ameliorative effect of DNE on cholesterol metabolism disorders in NAFLD mice *via* dual modulation of *FXR* and *LXR* is also reflected in other aspects. Hepatic *FXR* activation increased the expression of *Baat* and *Bacs* and elevated levels of conjugated BAs in the liver, indicating that DNE promotes cholesterol conjugation with amino acids, enhancing BA hydrophilicity for subsequent excretion into the gallbladder. Upregulation of *Bsep* and increased total BA levels suggest that DNE enhanced biliary BA transport, preventing cholestasis induced by elevated BA synthesis. Decreased expression of *Oatp* and *Ntcp* indicated that DNE may restrict BA reuptake into the liver *via* the enterohepatic circulation. Activation of intestinal *FXR* led to significant downregulation of the downstream target gene *Asbt*, upregulation of *Ostα*, and a marked increase in total BAs in ileal contents. This suggests that DNE reduces BA reabsorption in intestinal epithelial cells and promotes BA excretion from enterocytes, thereby mitigating enterotoxicity caused by BA retention. Furthermore, upregulation of *Abcg5/8* in the liver (downstream targets of *LXR*) and downregulation of *Npc1l1* in the intestine (another *LXR* target) indicate that DNE may limit dietary cholesterol absorption and promote hepatic cholesterol efflux, thus preventing further cholesterol accumulation in the body.

To validate the hypothesis that DNE ameliorates cholesterol metabolism disorders by dual regulation of *FXR* and *LXR*, we performed experiments on a cholesterol-accumulating HepG2 cell model. The results demonstrated that 100 nM DNE reduced the level of TC in hepatocytes, alleviated cholesterol accumulation, and significantly upregulated the expression of CYP7A1, C*YP27A1*, *FXR*, *SHP*, and *LXRα*, which was consistent with the results of *in vivo* experiments. Compared with the DNE and CDCA co-treatment group, the inductive effect of DNE on *SHP* was weakened, while that on *CYP7A1* was enhanced in the DNE alone group; compared with the DNE and GS co-treatment group, the inductive effect on *SHP* was enhanced and the inductive effect on *CYP7A1* was weakened in the DNE group, indicating that DNE can downregulate the expression of *CYP7A1* through the *FXR-SHP* pathway. On the other hand, the induction of *CYP7A1* was weakened in the DNE group compared with the DNE and GW co-treatment group, and the induction of *CYP7A1* was enhanced in the DNE group compared with the DNE and SR co-treatment group, indicating that the expression of *CYP7A1* was upregulated by DNE through *LXRα*. In conclusion, DNE regulates the expression of *FXR* and *LXRα*, and the overall effect on *CYP7A1* regulation was upregulation, thereby promoting the conversion of cholesterol to BAs and reducing cholesterol accumulation in hepatocytes. We also noted that the basal expression level of CYP7A1 in HepG2 cells is relatively low and that pharmacological agonists/antagonists may be subject to off-target effects; therefore, future studies employing primary mouse hepatocytes and loss-of-function approaches (e.g., siRNA-mediated silencing or genetic knockout of FXR and LXRα) are warranted to validate these findings and definitively establish pathway dependency. Additionally, we acknowledge that direct measurements of FXR/LXR transcriptional activity (e.g., luciferase reporter assays) were not performed in this study, and our conclusions regarding pathway modulation are based on changes in downstream gene expression and pharmacological interventions.

In summary, this study preliminarily verified that DNE restricts the absorption of exogenous cholesterol through *FXR/LXR*, enhances the biotransformation of cholesterol to BAs, promotes the transport of cholesterol and BAs, reduces the reuptake of BAs, and improves the cholesterol metabolism disorders in NAFLD mice. One advantage of *D. nobile Lindl.* as a therapeutic agent for NAFLD is its ability to compensate for the limitations of single-target drugs through the superposition of multiple pharmacological effects; however, cell experiments cannot fully simulate the complex internal environment of the body, and whether the level of oxysterols, the endogenous ligands of *LXRα*, increases significantly after DNE administration still needs more experimental data to be answered. Meanwhile, direct measurements of cholesterol flux (e.g., isotope tracing or CYP7A1 enzymatic activity) would provide more definitive evidence for enhanced cholesterol conversion in future investigations. Furthermore, as only endpoint measurements were performed, it remains unclear whether FXR and LXRα activation occur concurrently or sequentially; time-course experiments would be needed to resolve this temporal hierarchy.

## Data Availability

The raw data supporting the conclusions of this article will be made available by the authors, without undue reservation.
